# Multicentre Analysis of Cost, Uptake and Safety of Canadian Multidisciplinary Pancreatic Cyst Guidelines

**DOI:** 10.1093/jcag/gwad001

**Published:** 2023-02-28

**Authors:** Kevin Verhoeff, Alexandria N Webb, Daniel Krys, Danielle Anderson, David L Bigam, Christopher I Fung

**Affiliations:** Department of Surgery, 2D2 Walter Mackenzie Health Sciences Centre, University of Alberta, 8440 - 112 Street, Edmonton, Alberta T6G 2B7, Canada; Department of Surgery, McMaster University, Section B3 – Room 143 Juravinski Hospital, 711 Concession Street Hamilton, Ontario L8V 1C3, Canada; Faculty of Medicine and Dentistry, University of Alberta, 8440 - 112 Street Edmonton, Alberta T6G 2B7, Canada; Department of Laboratory Medicine and Pathology, 4B1.19 Walter Mackenzie Health Sciences Centre, University of Alberta, 8440 - 112 Street Edmonton, Alberta T6G 2B7, Canada; Department of Surgery, 2D2 Walter Mackenzie Health Sciences Centre, University of Alberta, 8440 - 112 Street, Edmonton, Alberta T6G 2B7, Canada; Department of Radiology, 2A2 Walter Mackenzie Health Sciences Centre, University of Alberta, 8440-112 Street Edmonton, Alberta T6G 2B7, Canada

**Keywords:** *Cost savings*, *Guidelines*, *MRI*, *Pancreatic cyst*, *Surveillance*

## Abstract

**Background:**

Pancreatic cystic lesions (PCLs) are common, with several guidelines providing surveillance recommendations. The Canadian Association of Radiologists published surveillance guidelines (CARGs) intended to provide simplified, cost-effective and safe recommendations. This study aimed to evaluate cost savings of CARGs compared to other North American guidelines including American Gastroenterology Association guidelines (AGAG) and American College of Radiology guidelines (ACRG), and to evaluate CARG safety and uptake.

**Methods:**

This is a multicentre retrospective study evaluating adults with PCL from a single health zone. MRIs completed from September 2018–2019, one year after local CARG guideline implementation, were reviewed to identify PCLs. All imaging following 3–4 years of CARG implementation was reviewed to evaluate true costs, missed malignancy and guideline uptake. Modelling, including MRI and consultation, predicted and compared costs associated with surveillance based on CARGs, AGAGs and ACRGs.

**Results:**

6698 abdominal MRIs were reviewed with 1001 (14.9%) identifying PCL. Application of CARGs over 3.1 years demonstrated a >70% cost reduction compared to other guidelines. Similarly, the modelled cost of surveillance for 10-years for each guideline was $516,183, $1,908,425 and $1,924,607 for CARGs, AGAGs and ACRGs respectively. Of patients suggested to not require further surveillance per CARGs, approximately 1% develop malignancy with fewer being candidates for surgical resection. Overall, 44.8% of initial PCL reports provided CARG recommendations while 54.3% of PCLs were followed as per CARGs.

**Conclusions:**

CARGs are safe and offer substantial cost and opportunity savings for PCL surveillance. These findings support Canada-wide implementation with close monitoring of consultation requirements and missed diagnoses.

## Teaser

Pancreas cysts are common and have a small chance of becoming cancer. The Canadian guidelines (CARGs) help doctors decide how and when patients should be monitored. This study looks at the cost of CARGs compared to other guidelines. It also looks at the safety of CARGs and if they are easy to use. This study looked at people with pancreatic cysts. After 3–4 years of following CARGs to monitor those people, the costs, missed cancers and guideline use was studied. We studied 1001 people and after using the CARGs for 3.1 years there was a big cost savings. For people that CARGs say don’t need monitoring, only 1% end up having cancer and most of those would not benefit from surgery. CARGs are safe and have cost savings. This study supports using CARGs across Canada.

## Graphical Abstract

**Figure:**
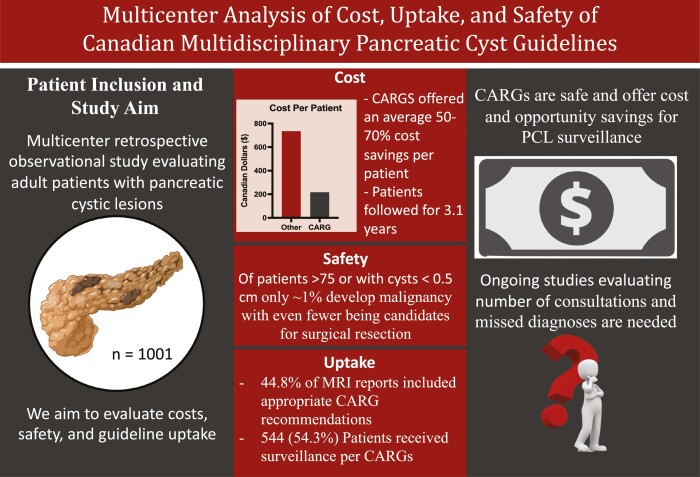
Verhoeff et al. Journal of the Canadian Association of Gastroenterology, 2023, 6, 86–93

## INTRODUCTION

With increasing cross sectional imaging, the prevalence of incidental pancreatic cystic lesions (PCLs) continues to rise, occurring in 2%–45% of imaging studies (1–6). While the risk of developing invasive neoplasia from PCLs remains low at 0.72% per year (5,7,8), pancreatic malignancy is 19.64 times higher in patients with any PCL (9). Surveillance guidelines provide recommendations to inform further evaluation and intervention (5,10). However, guidelines remain primarily based on expert opinion, due to limited evidence about the natural history of PCLs (2,4,11). This has led to development of multiple guidelines with variable adherence (4,12). Previously published North American guidelines include the American Gastroenterology Association guidelines (AGAG) and the American College of Radiology guidelines (ACRG) (13,14). The Canadian Association of Radiologists Incidental Findings Working Group recently published Multidisciplinary Pancreatic Cyst Guidelines (CARG) intended to simplify recommendations, improve adherence and create cost-effective guidelines without compromising safety (15). Notable changes within CARGs include stopping surveillance in patients over age 75 and for low risk PCLs <0.5 cm (15). Other key aspects of all three guidelines are summarized in Table 1. Despite CARG publication, no study exists evaluating their relative costs, safety or uptake.

**Table 1.** Summary of key features for three North American guidelines for surveillance of pancreatic cysts

Guideline	Indications for surgical consultation	Surveillance imaging recommendations	Indications for gastroenterology consultation	Key features or differences
**CARG**	Enhancing mural nodules Main pancreatic duct ≥10 mm Obstructive jaundice with cystic lesion in the pancreatic head	Follow up MRI at 1 year, repeat every 2 years. If cyst develops concerning features or grows ≥3 mm suggest gastroenterology consultation Discontinue follow up after 5-years if cyst unchanged or 10-years for patients aged 40–49	Age <40 Cyst ≥2.5 cm Focal wall thickening on cyst Non-enhancing mural nodules Main pancreatic duct 5–9 mm Regional pathologic lymph node enlargement Abrupt change in pancreatic duct diameter with upstream atrophy Cyst wall enhancement	Simple cystic lesions 5 mm in size do not require follow-up Patients >75 years old do not require follow up Unique guidelines for patients <40 years old and 40–49 years old
**AGAG**	Presence of both a solid component and a dilated pancreatic duct concerning features on EUS and FNA	Follow up MRI at 1 year, repeat every 2 years. If cyst changes suggest gastroenterology consultation Discontinue follow up after 5-years if cyst unchanged	Cysts with ≥2 high risk features including: Cyst ≥3 cm Presence of a solid component Dilated main pancreatic duct	Discontinue surveillance if “the patient is no longer a surgical candidate”
**ACRG** ^*^	Cyst ≥2.5 cm with interval growth or high risk features including: Obstructive jaundice Enhancing solid component Dilated main pancreatic duct ≥10 mm	Generally follows cysts for 9 or 10-years If cyst changes suggest gastroenterology consultation Imaging interval ranging from q6 months to q2 years based on patient and cyst factors	Cyst ≥2.5 cm Offers EUS/FNA as an alternative to imaging surveillance for cysts 1.5–2.5 cm	Unique recommendations for patients ≥80 years old Unique recommendations based on patient age, cyst size and cyst features

^*^Summary of the ACRG guidelines represents only one of several options presented in the guideline.

CARG, Multidisciplinary Pancreatic Cyst Guidelines; AGAG, American Gastroenterology Association Guidelines; ACRG, American College of Radiology Guidelines; EUS, endoscopic ultrasound; FNA, Fine needle aspiration.

The most common neoplastic PCL are intraductal papillary mucinous neoplasms (IPMNs), which have up to a 2%–44% rate of malignant transformation (2,5,10). High risk PCL features including solid components, enhancing mural nodules, jaundice and pancreatic duct dilation of ≥10 mm can inform surveillance decisions (3,16). All current PCL guidelines recommend magnetic resonance imaging (MRI) for surveillance (1–6,17). Following MRI, guidelines outline surveillance imaging intervals or suggest gastroenterology consultation for endoscopic ultrasound (EUS) with fine needle aspiration (FNA), or surgical consultation (13,18). However, each surveillance decision affects guideline costs and accuracy. Determining whether recently published CARGs offer cost savings, while remaining safe and useable, is crucial to inform uptake and optimization. 

We aim to evaluate the cost of PCL surveillance using CARGs compared to other North American guidelines. Additionally, we characterize the safety and uptake of CARGs following implementation within a multi-centre health zone.

## METHODS

### Study Design and Formulation of Research Question

This is a multi-centre retrospective study evaluating cost, safety and uptake of recently published CARGs. We included adults diagnosed with PCL from a single health zone with a population >1.3 million serviced by four hospital-based imaging services. The health zone’s patients represent a broad population including mixed rural and urban patients. Outcomes include assessment of costs for those receiving surveillance according to CARGs compared to other guidelines, uptake of CARGs and safety of CARGs following implementation as a zone guideline. Further, we provide a model to predict and compare cost of PCL surveillance using North-American guidelines including CARGs, AGAGs and ACRGs to contextualize economic findings of this study. The study has been reviewed and approved by research ethics board (Pro00090110).

### Patient Inclusion, Study Protocols and Outcome Variables

All patients ≥18 years old with PCLs in the health zone were included. Patients were identified by screening all abdominal MRI scans completed within the health zone from September 1, 2018 to September 1, 2019. This represents a 12-month sample of convenience after CARG implementation as a zone guideline in 2017, prior to their publication in 2021 (15). MRI scans were identified using the zone’s imaging software with an advanced search limited by date and imaging modality (i.e., MRI).

Following identification of patients with PCL, the following data was extracted from the patient record: patient age at study date, if the PCL was an incidental finding or a follow-up scan, the largest PCL size, number of PCLs, presence of any high risk or concerning features as per the CARGs, and inclusion of a correct CARG recommendation. All subsequent surveillance after PCL identification was reviewed to evaluate CARG costs, adherence and incidence of malignancy in patients with suggested screening discontinuation per CARGs (i.e., those with cysts <0.5 cm or age >75). Finally, using demographics and cyst features at time of PCL identification, projected cost of implementing CARG, AGAG and ACRGs for 10-years was calculated using a cost model detailed below.

The primary outcome of this study was the cost of surveillance for patients included in this study who were followed according to CARGs compared to those receiving surveillance outside of CARG recommendations. To accomplish this, surveillance for patients was evaluated from time of inclusion (2018–2019) until May 1, 2022 with all MRIs and hospital visits within the province reviewed. We extracted the reason for MRI, imaging findings including interval cyst growth and categorized follow-up as CARG-compliant versus non-compliant. A grace period of ±3 months at each required scan or consultation was allowed to account for scheduling variation. In all cases, if PCLs developed concerning features or grew larger than >2.5 cm, a referral to GI or to surgery was required to be deemed appropriate. Patient follow-up was censored at the time of mortality, diagnosis of pancreatic malignancy or PCL surgery. The number and cost of all MRIs and consultations for patients followed according to CARGs were compared to patients receiving surveillance according to other recommendations. This allowed for costs to consider interval cyst change and rate of surgical intervention between groups to assess the validity of our cost model. Patients lost to follow-up immediately following their initial MRI scan identifying PCL were excluded from this analysis. Costs of imaging and consultation were calculated as detailed in the cost assumptions section below.

Secondary outcomes from the study included safety and uptake of CARGs. In particular, we aimed to assess the safety of discontinuing surveillance for patients with PCL <0.5 and age >75. To assess safety, imaging from patients with discontinuation of PCL screening according to CARGs was evaluated with identification of any new pancreatic malignancy. All imaging reports after PCL identification including MRI, computed tomography, ultrasounds and X-rays for included patients were reviewed until May 1, 2022. This methodology assumes that any patient developing pancreatic malignancy will undergo diagnostic imaging demonstrating disease. Any potential pancreatic malignancy was confirmed through chart review with case description to guide CARG evaluation. Uptake of CARGs was evaluated by the proportion of patients receiving surveillance according to CARGs, and the proportion of initial MRIs with appropriate recommendations within the imaging report.

In addition to reporting the cost of surveillance for those followed according to CARGs compared to other guidelines, we provide a cost model to evaluate the predicted cost difference of CARGs compared to other North American guidelines including AGAGs and ACRGs. Using the identified patient demographics and cyst features, a cost model was developed applying guidelines to calculate the number of surveillance MRIs, gastroenterology consultations and surgical consultations based on 100% adherence to each guideline over a 10 year period (13–15).

### Cost Calculation and Model Assumptions and Health Zone Imaging Protocols

For both real cost calculation and modelling the total costs for an MRI was $319.70, which includes the cost of radiologist consultation and contrast. Total cost of gastroenterology consultations ($780) included the completion of consultation ($185), physician fees for completion of an EUS ($200), and FNA ($100) and cost of FNA needle ($295) while the cost per surgical consultation was $185. Breakdown of costs for imaging and consultation including the source of cost data can be found in Supplementary Material Table S1.

Cost modelling was based on the following assumptions. First, cost for all MRIs included the cost of contrast. Although a growing body of evidence suggests that follow-up MRIs can be completed without contrast (19), that practice remains heterogeneous and it was assumed that rates of contrast use would be equal between groups enabling a relative comparison. Secondly, although AGAGs only provide five-year follow-up recommendations, the same recommendations were applied over ten years to allow comparison to CARGs and ACRGs. To enable equal cost analysis, adherence to the guidelines was assumed to be 100% and all calculations were completed assuming no interval growth of the PCL. This assumption has been applied to all guidelines and enables an equal comparison.

Typical PCL MRI surveillance included multiplanar T1 and T2 weighted sequences, diffusion weight imaging sequences, an MRCP sequence, and pre/post-gadolinium T1 fat saturated sequences. The routine pancreatic cyst follow-up protocol includes axial T1 and T2 weight sequences and a MRCP sequence.

### Statistical Analysis

Statistical analysis was performed using STATA 17 (StataCorp, College Station, TX, USA). Categorical data were expressed as percentages, while continuous data were expressed as mean ± standard deviation. Univariate analysis using chi-squared for categorical data and ANOVA for continuous data was performed to determine between group differences. Cost outcomes are presented in 2020 Canadian dollars without statistical comparison cost data due to lack of technical reliability (20).

## RESULTS

A total of 6698 abdominal MRIs were completed in the zone with 1001 (14.94%) identifying PCL, of those 426 (42.6%) were for PCL follow-up. In patients with identified PCLs, average age was 64.4 (SD 12.01), the average cyst size was 1.24 cm (SD 1.105 cm), concerning features were found in 42 MRIs (Table 2).

**Table 2.** Demographics of patients undergoing MRI abdomen and details of identified pancreatic cysts at baseline

Number of patients/MRI with PCL	1001
Age	64.4 (12.01)
Age categories (%)	
<40	43 (4.32%)
40–50	79 (7.93%)
51–75	708 (71.08%)
>75	166 (16.67%)
Cyst size (SD)	1.24 cm (1.105 cm)
Number of cysts (%)	
1	532 (53.41%)
2	125 (12.55%)
3	32 (3.21%)
≥4	307 (30.82%)
MRIs with high risk features (%)	42 (4.19%)
MRIs with concerning Features (%)	35 (3.50%)

Continuous data are presented as means (standard deviation) and categorical as absolute values (percentages).

MRI, Magnetic resonance imaging; PCL, Pancreatic Cystic Lesion.

### True Cost Comparison of CARGs Compared to Alternative Surveillance Methods

Following identification of 1001 patients with PCLs from initial MRIs completed between 2018 and 2019 there were 544 (54.3%) patients followed according the CARGs, 241 (23.9%) followed according to other guidelines and 216 (21.6%) patients lost to follow-up. Surveillance for the 241 patients followed outside of CARGs was heterogenous with intermittent use of AGAGs, ACRGs, Fukuoka guidelines and intermixed guideline application.

In terms of demographics and cyst characteristics (Table 3), patients followed according to CARGs were older than others with surveillance (65.9 ± 13.6 CARG vs. 62.4 ± 10.1 other, *P* < 0.001). However, when excluding patients >75 (*n* = 159) to account for patients that were lost to follow-up and were inadvertently categorized as being followed per CARGs, age of the two groups was similar (60.9 ± 11.6 CARG with *n* = 413 vs. 60.2 ± 8.4 other with *n* = 214, *P* = 0.40). Both groups received a similar duration of follow up (1154.5 ± 120.6 days CARG vs. 1165.4 ± 104.0 days other, *P* = 0.22) equating to approximately 3.1 years. In terms of cyst features, patients followed per CARGs had smaller cysts (1.1 ± 1.1 cm CARG vs. 1.6 ± 1.1 cm other, *P* < 0.001) but after excluding patients with cysts <0.5 cm (*n* = 244), sizes were similar (1.6 ± 1.2 cm CARG with *n* = 322 vs. 1.7 ± 1.1 cm other with *n* = 220, *P* = 0.35). At onset of surveillance, cysts followed per CARGs had a higher rate of high risk features (3.1% CARG vs. 6.2% other, *p* = 0.042) but a similar rate of concerning features (5.7% CARGs vs. 7.9% other, *P* = 0.25) with Table 3 characterizing the specific features.

**Table 3.** Demographics and cyst characteristic comparing patients followed according to the Canadian Multidisciplinary Pancreatic Cyst Guidelines versus those followed according to other surveillance methods

	CARG	Other	*P*-value
Age	65.9 (13.6)	62.4 (10.1)	<0.001
Age categories			<0.001
<40	3 (1.2)	37 (6.8)	
40–50	82 (34.0)	107 (19.6)	
51–75	124 (51.5)	253 (46.4)	
>75	32 (13.3)	148 (27.2)	
Follow-up duration (days)	1154.5 (120.6)	1165.4 (104.0)	0.22
**Cyst features**			
Cyst size (cm)	1.1 (1.1)	1.6 (1.1)	<0.001
High risk features	17 (3.1)	15 (6.2)	0.042
Characteristics of high risk features			
Obstructive jaundice	2 (0.4)	1 (0.4)	0.92
Enhancing solid component	10 (1.8)	13 (5.4)	0.006
Main pancreatic duct ≥10 mm	6 (1.1)	3 (1.2)	0.86
Concerning features	31(5.7)	19 (7.9)	0.245
Characteristics of concerning features			
Thickened walls	1 (0.2)	0 (0)	0.51
Non-enhancing solid component	0 (0)	2 (0.8)	0.033
Main pancreatic duct 5–9 mm	22 (4.0)	15 (6.2)	0.18
Lymphadenopathy	7 (1.3)	1 (0.4)	0.26
**Surveillance**			
MRIs per patient	0.4 (0.7)	1.8 (1.0)	<0.001
Gastroenterology consults	51 (9.4)	41 (17.0)	0.002
Surgery consults	48 (8.8)	35 (14.5)	0.016
Pancreatic surgical resection	16 (2.9)	8 (3.3)	0.77

Demographics and cyst characteristics after exclusion of patients with cysts <0.5 cm and >75 years old are presented in Supplementary Material Table S2.

Continuous data are presented as means (standard deviation) and categorical as absolute values (percentages) with *P*-values resulting from analysis using chi-squared for categorical data and ANOVA for continuous data.

CARG, Multidisciplinary Pancreatic Cyst Guidelines; MRI, magnetic resonance imaging.

In terms of resource utilization, patients followed per CARGs received significantly fewer MRIs per person compared to other guidelines (0.4 ± 0.7 CARGs vs. 1.8 ± 1.0 other, *P* < 0.001, Table 3). Significantly fewer patients followed with CARGs were discovered to have interval cyst growth during surveillance (1.1% CARG vs. 20.3%, *P* < 0.001). Accordingly, the rate of consultation was lower in patients followed per CARGs than other guidelines (9.4% CARG vs. 17.0% other, *P* = 0.002). Similarly, rate of surgical consultations was lower than other guidelines (8.8% CARG vs. 14.5% other, *P* = 0.016). Despite these differences, the rate of PCL surgical resection in both groups was similar (2.9% CARG vs. 3.3% other, *P* = 0.77, Table 3). When applying costs to these resources, the cost per person of MRIs ($127.88 CARG vs. $575.46 other), gastroenterology consultations ($73.12 CARG vs. $132.68 other) and surgical consultations ($16.32 CARG vs. $26.87 other) were all lower using CARGs compared to other guidelines. Average total costs per person followed with CARGs was lower than other surveillance methods ($217.32 CARG vs. $735.01 other) and represented a 70.4% cost savings.

To exclude patients with cysts <0.5 cm and age >75 who may have been lost to follow-up and potentially incorrectly categorized as being followed according to CARGs, a subgroup analysis excluding all patients with cysts <0.5 cm AND age >75 was completed. Following exclusion there were 418 patients, with 225 (53.8%) followed per CARGs and 193 (46.2%) followed using other recommendations. Age, duration of follow-up, cyst size and rate of high risk or concerning cyst features were all similar (Supplementary Material, Table S2). There remained fewer MRIs per patient with CARGs (0.8 ± 0.9 CARG vs. 1.8 ± 1.0 other). For these patients, rates of gastroenterology consultation (17.9% CARG vs. 20.7% other, *P* = 0.47), and surgical consultation (17.8% CARG vs. 13.0% other, *P* = 0.18) were not statistically different between groups (Supplementary Material, Table S2). Overall, cost of resources in this subgroup demonstrated a lower cost per person of MRIs ($255.76 CARG vs. $575.46 other) and gastroenterology consultation ($138.66 CARG vs. $161.66 other) for CARGs and a higher cost of surgical consultations ($32.89 CARG vs. $23.96 other) compared to those surveyed using other guidelines. Average total costs per person followed according to CARGs was lower ($427.31 CARG vs. $761.08 other) than other surveillance methods and represented a 56.1% cost savings.

### Adherence to CARGs

With regards to adherence, a total of 448 (44.8%) of the initially evaluated MRI reports contained appropriate follow up suggestions based on the CARGs, with substantial inter-hospital variability, including one site not including any CARG recommendations (Supplementary Material Table S3). As above, when evaluating subsequent imaging of initially identified patients, 544 (54.3%) received PCL follow-up as per CARGs. Therefore, 457 (45.7%) of patients received PCL follow-up outside of CARG recommendations; this includes 159 (36.6%) under screened patients and the remaining patients being over-screened with an average of 2.2 (SD 1.8) MRIs. Of the 253 patients aged >75 with PCLs, 47 (18.6%) received follow-up MRIs outside of CARG recommendations with those patients receiving on average 2.4 (SD 2.2) MRIs during the study period. Similarly, 40 (13.4%) patients with PCLs <0.5 cm received repeat MRIs outside of CARG recommendations.

### Safety of CARGs

With regards to safety we identified 5 patients who developed pancreatic malignancy out of 419 suggested to have screening discontinuation as per CARGs. One patient had discontinued follow up for PCL <0.5 cm; this 54-year-old female patient had a history of chronic recurrent pancreatitis leading to computed tomography one year after PCL diagnosis, demonstrating metastatic pancreatic adenocarcinoma. In patients with suggested discontinuation of surveillance due to age >75 (*n* = 253) only 4 (1.6%) had subsequent diagnosis of pancreatic adenocarcinoma, with only one patient presenting with metastatic disease. This patient had a previous diagnosis of cirrhosis and previously been deemed too high risk for an inguinal hernia repair and cholecystectomy >1 year prior to PCL diagnosis. Three others were followed with repeat MRI beyond guideline recommendations, which identified their malignancy. Of these three patients, one 92-year-old male had previously received surgical consultation for their high risk PCL in 2016 and decided against pancreatic resection due to severe chronic obstructive pulmonary disease and frailty, one 86-year-old female was offered surgical resection but elected to proceed with palliative therapy, and one 83-year-old male initially refused surgery but elected for a distal pancreatectomy after further cystic growth. Overall, of patients >75 years of age with PCL (*n* = 253), continued surveillance could have theoretically altered care in 0.4% (*n* = 1) of patients. In patients with PCL <0.5 cm (*n* = 299), continued surveillance could have theoretically altered care in 0.3% (*n* = 1) patient. Notably, in both circumstances patients received repeat surveillance outside of guidelines without delayed diagnosis.

### Predicted Cost Comparison of Guidelines Applied Over Ten Years

Modeling CARG application to identified patients predicted 35 surgical consultations, 119 gastroenterology consultations and 1304 MRIs over ten years of surveillance, yielding a predicted total cost of $516,183 (Table 4). The CARGs had the lowest number of predicted MRIs over ten years with a cost of $416,888 (Table 4). The CARGs do not complete any further surveillance for patients over the age of 75 or for cysts smaller than 0.5cm; 419 (41.86%) MRIs met these criteria, which would cost $133,954.30 if completed. The predicted cost for surgical consultation for the CARGs was between AGAG and ACRGs, costing $6,475. The predicted cost for gastroenterology consultation when following CARGs was the highest and cost $92,820. Taking into consideration all costs, the predicted expense reduction over ten years between the CARGs and AGAGs is $1,392,242 (a 73.0% cost reduction) and between the CARGs and ACRGs is $1,408,424 (a 73.2% cost reduction).

**Table 4.** Predicted number and cost of surgical consultations, gastroenterology consultations and MRI scans over a ten-year surveillance period

	CARG	AGAG	ACRG
Surgery	35	4	76
Surgery cost (CAD$)	6475	740	14,060
Gastroenterology	119	48	82
Gastroenterology cost (CAD$)	92,820	37,440	63,960
MRI	1304	5850	5776
MRI cost (CAD$)	416,888	1,870,245	1,846,587
**Total cost (CAD$)**	**516,183**	**1,908,425**	**1,924,607**

Costs shown here are predicted after applying the CARG, AGAG and ACRGs to 1001 patients within a single health zone assuming no cyst growth, no further cyst incidence in the population and costs determined as per Supplementary Material Table S1.

CARG, Multidisciplinary Pancreatic Cyst Guidelines; AGAG, American Gastroenterology Association Guidelines; ACRG, American College of Radiology Guidelines.

Modelling surveillance using the AGAGs predicted 4 surgical consultations, 48 gastroenterology consultations and 5850 MRIs over ten years of surveillance, yielding a predicted cost of $1,908,425 (Table 4). Associated MRI costs were predicted to be $1,870,245, which represents a predicted additional cost of $1,453,356 compared to CARGs over ten years (Table 4). The predicted cost of surgical consultation was $740, a $5,735 reduction compared to CARGs and the cost of gastroenterology consultation $37,440, offers a potential $55,380 reduction.

Modelling surveillance using the ACRGs predicted 76 surgical consultations, 82 gastroenterology consultations and 5776 MRIs over ten years of surveillance, yielding a predicted cost of $1,924,607 (Table 4). Modelling ACRGs predicted an MRI cost of $1,846,587, which is $1,429,698 more than predicted costs for CARGs (Table 4). When following ACRGs, predicted cost of surgical consultation was $14,060, which is $7585 higher than when following CARGs. However, the predicted cost of gastroenterology consultation was $63,960, a potential $28,860 reduction compared to CARGs.

## DISCUSSION

Compared to other North-American PCL surveillance guidelines, the CARGs offer substantial cost savings yet remain usable and safe. Cost assessment demonstrated a 50%–70% cost savings compared to other guidelines after following patients for >3 years, with modelling further supporting cost benefits suggesting >70% potential savings compared to other commonly used North American guidelines. Providers should be reassured, and take into consideration when selectively screening patients with cysts <0.5 cm and age >75, that only ~1% develop malignancy with even fewer being candidates for surgical resection. This data should support further CARG uptake, and inform future guideline optimization.

With regards to health care resource utilization this represents the first study evaluating cost savings of CARGs and differences between North American pancreatic cyst surveillance guidelines. Cost savings when applying CARGs were dramatic in just 3 years, with absolute cost savings expected to be substantial if CARGs are applied across several health zones. Modelling to compare to other North American guidelines show similar cost benefits further supporting CARG application. While our model was simplistic with limitations discussed below, both real and predicted cost remained 50%–70%, indeed, highlighting a strength of CARGs. Broad provincial and national use should be encouraged and could lead to important cost reduction without compromised patient care. Beyond the cost savings associated with CARG use, both our real data and model demonstrate markedly fewer MRIs required due to discontinuation of follow up in low risk patients and those unlikely to recognize benefits from surveillance (15). In 2018 the waiting list for a MRI in our province was 57,234 people (21). Given this large waiting list, a decrease in the number of abdominal MRIs also allows for better access to MRI, which is likely to help reduce this waiting list.

Despite decreased costs and increased potential MRI access, an important difference between true costs and our model should be highlighted. Our model predicted that CARGs may produce comparatively more surgical consultations than the AGAGs, and substantially more gastroenterology consultations than both the AGAGs and ACRGs. While the absolute PCL referral number still remains small, increased surgical consultation volume may be noted for hepatobiliary surgeons, of which only 52 existed in Canada as of 2015, with 30.1% stating that too few hepatobiliary specialists existed in their region (22). For both surgical and gastroenterology specialists, these additional consultations may increase their workload and prolong consultation waitlists. However, real data did not show significant differences in rates of consultation for CARGs compared to other surveillance methods. This may occur because of more MRIs being completed with other guidelines, leading to discovery of interval cyst growth and ensuing surgical or gastroenterological consultations. However, considering similar rates of surgery, the clinical significance of discovering these cyst developments is unclear. While future studies evaluating hepatobiliary and gastroenterology PCL consultations and their effect on wait lists may be required, this current data is reassuring and may even provide further support for CARGs.

Beyond improved health care utilization, this is the first study providing preliminary safety data for CARG recommendations to discontinue PCL surveillance in patients >75 or with PCLs <0.5 cm. We provide 3–4 year follow up for >1000 patients and demonstrate that long-term outcomes are largely unaltered in patients with discontinued surveillance. However, it should be highlighted that for patients >75 who may benefit from pancreatic cancer therapies, or patients with unique clinical scenarios (exemplified by our patient with chronic pancreatitis) ongoing surveillance should be considered. However, when recommending surveillance for these patients physicians should be mindful of an extremely low malignancy rate ~1% and even lower rate of altering care. Additionally, the relatively short safety follow-up period of this study, especially in light of relatively slow growing nature of these cysts, highlights that specialists including gastroenterology and surgery involved in management of pancreatic malignancy should continue to highlight patients outside of CARG recommendations who may have benefited from ongoing surveillance.

Finally, with regards to guideline use, it appears that CARGs have relatively strong uptake but have ongoing opportunity for further education and dissemination. In this study 44.8% of MRIs included suggested CARG recommendations. Previous literature found that only 23.7% of reports with PCLs included a recommendation (12). Inclusion of recommendations within a radiology report has previously been associated with PCL follow-up with an odds ratio of 5.5, with 83.0% of patients receiving follow-up having an imaging report with radiology recommendations. In fact, nearly 60% of patients in this study received surveillance per CARGs. Considering these guidelines were implemented locally and remain relatively novel, we suspect that CARG recommendation inclusion in reports and uptake will continue to increase. Ongoing work on education for physicians and residents involved with ordering, interpretating and consultations will be important for uptake. This should include providers from family medicine, gastroenterology, hepatobiliary surgery and radiology.

Despite these findings, limitations of this study should be highlighted. Firstly, this study only compares cost to other North American based guidelines, yet other important guidelines exist and may also be applied in North America. Our cost model also has several assumptions including no PCL growth over time and 100% adherence; in clinical practice some cysts may grow leading them to meet CARG criteria for consultation or surveillance, which may reduce cost savings demonstrated here. Additionally, 100% adherence is unlikely achievable and only allows relative cost comparison of guidelines, with true cost savings likely less than reported here. The costs also do not take into consideration the cost of surgical intervention if it is deemed appropriate after a surgical consultation, nor do they consider repeat follow-ups or additional testing ordered after consultation. Despite these limitations of our model, the real data does support cost savings and should lend support for our conclusions. This study also represents a retrospective review with sample size based on convenience; however, we believe the study duration is adequate to capture a representative sample of MRIs in this health zone. A substantial proportion of the data was also collected during the coronavirus-19 worldwide pandemic, which could have affected recommendations provided by specific providers and has an uncertain effect on our outcomes. Additionally, while we have provided 3–4 year uptake and safety data for the included patients, ongoing evaluation of guideline uptake and safety are needed. Natural history for pancreatic cysts typically involves slow growth or malignant transformation over many years and it is possible that an increasing number of missed malignancy will present in our cohort of patients over time. Follow-up studies evaluating long-term safety are planned, with additional evaluation of uptake following recent CARG publication. Canadian wide awareness regarding the potential for missed malignancy, especially in patients who may benefit from operative intervention aged >75 should be highlighted and continuously evaluated over time. This study also does not account for how many incidental PCL will be found on a yearly basis and the further surveillance or consultations that would be required. Future studies evaluating the impact of these guidelines on surgical and gastroenterological consultation will be required. Finally, this study is based in a Canadian setting where health care is primarily publicly funded, which may not be applicable to other health care systems.

## CONCLUSION

The CARGs represents a Canadian specific guideline for PCL surveillance that, compared to other North American guidelines, offer substantial cost savings and potentially improved MRI access. Current follow-up data supports safety of current CARG recommendations to discontinue surveillance for patients >75 years old and with PCL <0.5 cm, and also suggests ease of CARG uptake, however, future studies evaluating long-term safety are needed.

## Supplementary Material

gwad001_suppl_Supplementary_Material

## Data Availability

The data underlying this article cannot be shared publicly to protect the privacy of individuals that participated in the study. The data will be shared on reasonable request to the corresponding author.
