# Supplementary material for: Multicentre Analysis of Cost, Uptake and Safety of Canadian Multidisciplinary Pancreatic Cyst Guidelines
Source: J Can Assoc Gastroenterol. 2023 Feb 28;6(2):86–93. doi: 10.1093/jcag/gwad001 (PMC10071295; doi:10.1093/jcag/gwad001)
Supplement: gwad001_suppl_Supplementary_Material [file gwad001_suppl_supplementary_material.docx]

**Supplementary Material**

**Table S1:** Cost breakdown and source of costs for magnetic resonance imaging, gastroenterology consultation, and surgical consultation

| Procedure or Consultation Type | Breakdown of Cost and Total Cost ($CAD) | Source of Cost Data |
| --- | --- | --- |
| MRI | Consultant Fee: 218.10  Contrast: 101.60  **Total: 319.70** | Alberta Medical Association Fee Guideline and Health Zone Department of Radiology Costs |
| Surgical Consultation | Consultant Fee: 185.00  **Total: 185.00** | Alberta Medical Association Fee Guideline |
| Gastroenterology Consultation | Consultant Fee: 185.00  EUS Physician Fee: 200.00  FNA Physician Fee: 100.00  FNA needle: 295  **Total: 780.00** | Alberta Medical Association Fee Guideline  FNA needle cost supplied by the Department of Gastroenterology Costs |

MRI: magnetic resonance imaging; EUS: Endoscopic ultrasound; FNA: Fine needle aspirate

**Table S2:** Demographics and Cyst Characteristic Comparing Patients Followed According to the Canadian Multidisciplinary Pancreatic Cyst Guidelines versus those followed according to other surveillance methods after exclusion of patients with cyst <0.5 cm and age >75.

|  | CARG  n =225 | other  n =193 | p-value |
| --- | --- | --- | --- |
| Age | 61.0 (12.7) | 60.6 (8.4) | 0.17 |
| Follow-up Duration (days) | 1147.4 (117.3) | 1169.2 (104.0) | 0.05 |
| **Cyst Features** | | | |
| Cyst size (cm) | 1.6 (1.2) | 1.6 (0.9) | 0.96 |
| High risk features | 13 (5.8) | 12 (6.2) | 0.85 |
| Characteristics of high risk features |  |  |  |
| Obstructive jaundice | 1 (0.4) | 1 (0.5) | 0.91 |
| Enhancing solid component | 9 (4.0) | 11 (5.7) | 0.42 |
| Main pancreatic duct ≥10 mm | 3 (1.3) | 1 (0.5) | 0.39 |
| Concerning features | 20 (8.9) | 9 (4.7) | 0.09 |
| Characteristics of concerning features |  |  |  |
| Thickened walls | 1 (0.5) | 0 (0) | 0.35 |
| Non-enhancing solid component | 0 (0) | 2 (1.0) | 0.126 |
| Main pancreatic duct 5-9 mm | 15 (6.7) | 5 (2.6) | 0.052 |
| Lymphadenopathy | 4 (1.8) | 1 (0.5) | 0.24 |
| **Surveillance** | | | |
| MRIs per patient | 0.4 (0.7) | 1.8 (1.0) | <0.001 |
| Gastroenterology consults | 40 (17.9) | 40 (20.7) | 0.47 |
| Surgery consults | 40 (17.8) | 25 (13.0) | 0.18 |
| Pancreatic surgical resection | 14 (7.1) | 5 (2.6) | 0.062 |

Continuous data are presented as means (standard deviation) and categorical as absolute values (percentages) with p-values resulting from analysis using chi-squared for categorical data and ANOVA for continuous data.

CARG: Multidisciplinary Pancreatic Cyst Guidelines; MRI: magnetic resonance imaging

**Table S3**. MRIs with appropriate follow-up as per Multidisciplinary Pancreatic Cyst guidelines in the report by hospital

|  | MRIs | Report Includes Appropriate Follow-up Recommendation (%) |
| --- | --- | --- |
| Zone | 1001 | 448 (44.76) |
| Hospital A | 476 | 197 (41.39) |
| Hospital B | 314 | 141 (44.90) |
| Hospital C | 138 | 75 (54.35) |
| Hospital D | 73 | 0 (0) |
